# Cardiovascular health status between standard and nonstandard workers in Korea

**DOI:** 10.1371/journal.pone.0178395

**Published:** 2017-06-01

**Authors:** Jong Ju Seon, Yu Jin Lim, Hae Won Lee, Jae Moon Yoon, Sang June Kim, Seulggie Choi, Ichiro Kawachi, Sang Min Park

**Affiliations:** 1 Department of Family Medicine & Department of Biomedical Sciences, Seoul National University College of Medicine, Seoul, South Korea; 2 National Cancer Center, Goyang, South Korea; 3 Department of Radiation Oncology, Seoul National University Hospital, Seoul, South Korea; 4 Chungha Geriatric Hospital, GyeongJu, South Korea; 5 Department of Biomedical Sciences, Seoul National University Graduate School, 103 Daehakro, Jongro-gu, Seoul, South Korea; 6 Department of Social and Behavioral Science, Harvard School of Public Health, Boston, MA, United States of America; 7 Department of Global and Population Health, Harvard School of Public Health, Boston, MA, United States of America; University of Bristol, UNITED KINGDOM

## Abstract

**Objectives:**

The effect of employment insecurity on employee health is an important public health issue due to the recent effects of neoliberalism and the global financial crisis (2007–2008) on labor markets. This study aims to evaluate the differences in cardiovascular health status and the use of preventive screening services between standard and nonstandard workers.

**Methods:**

Waged employees (N = 5,338) between the ages of 20 and 64 were grouped into standard (full-time, permanent) and nonstandard (part-time, temporary, or daily) employees. Data from the Fourth Korea National Health and Nutrition Examination Survey, 2007–2009, a nationwide representative survey, were examined, including cardiovascular health risk behaviors (tobacco, alcohol, physical inactivity), measured morbidities (blood pressure, blood glucose level, lipid profiles, body mass index), and the use of screening services for hypertension and diabetes mellitus.

**Results:**

Female nonstandard employees tended to have higher blood pressure than did female standard employees (adjusted odds ratio, aOR 1.42, 95% confidence interval, CI 1.02 to 1.98). However, nonstandard employees (both men and women) were less likely to use preventive screening services for hypertension (aOR 0.72, 95% CI 0.54 to 0.94 in men; aOR 0.56, 95% CI 0.43 to 0.73 in women) and diabetes (aOR 0.58, 95% CI 0.43 to 0.79 in men; aOR 0.55, 95% CI 0.43 to 0.71 in women).

**Conclusion:**

Nonstandard work is associated with the underuse of screening services and poorer cardiovascular health in a specific population. Policies to reduce employment insecurity and encourage nonstandard employees to receive health screening services should be prioritized.

## Introduction

Following the 1997 financial crisis in Asia, significant changes have occurred in employment patterns as a result of neoliberalism and the increased demand for labor market flexibility. Beyond the dichotomous concepts of employment status (i.e., employed versus unemployed) [1], there has been an emergence of “nonstandard employment,” which includes working conditions outside of full-time and permanent employment [2]. A decade after the Asian financial crisis, the global financial recession of 2007–2008 further propagated the economy towards increased unemployment and employment insecurity [3–5].

It has been shown that employment and working conditions affect health status, with nonstandard employees experiencing more adverse effects on their physical and mental health compared to standard employees [6]. European studies published in the 2000s revealed that poor self-rated health, mental distress, and mortality rates increased with greater employment insecurity [7–9], and similar results have been reported in studies from the United States, Canada, and South Korea [10–13]. However, some studies have failed to show significant differences in cardiovascular health status between standard and nonstandard workers. For example, while several researchers have noted increased incidence or mortality from coronary heart disease in employees who are placed in precarious employment, organizational restructuring, downsizing or job insecurity [14–17], there has not been a definite and consistent association between measured cardiovascular risk factors and unstable employment conditions [18–19].

Although South Korea experienced an unprecedented increase in nonstandard work following the 1997–98 financial crisis combined with a poorly equipped social safety net (social insurance, pensions, fringe benefits, and job insurance), we were unable to find any systemic studies that examined the differences in individual health behaviors and outcomes between standard and nonstandard workers [13, 20]. Therefore we sought to examine the impact of nonstandard employment on employee health status using a nationwide representative survey. Specifically, we focused on assessing cardiovascular health behaviors, measured morbidities (blood pressure, blood glucose level, lipid profiles, body mass index), and the use of preventive screening services for hypertension and diabetes mellitus.

## Methods

### Data collection and study population

We used the Fourth Korean National Health and Nutrition Examination Survey (KNHANES IV), which was conducted by the Korean Ministry of Health and Welfare, for data collection. KNHANES IV used a stratified, multistage sampling design of 24,871 individuals in households across 600 national districts; sampling units were based on geographical area, gender, and age using household registries. The survey is composed of four parts: the Health Interview Survey; the Health Promotion Knowledge, Attitude, and Practice (HP-KAP) Survey; the Nutrition Survey; and the Health Examination Survey. The Health Interview Survey, Health Behavior Survey, and Nutrition Survey are self-administered questionnaires. The Health Examination Survey, consisting of measured variables such as blood pressure (BP), blood glucose level and body mass index (BMI), was conducted by trained nurses. As the survey data analyzed are publicly available, this study was waived by the Institutional Review Board of Seoul National University Hospital.

We performed cross-sectional analyses of data from 5,338 individuals who completed the Health Interview Survey, HP-KAP, and Health Examination Survey. The inclusion criteria included individuals who were: (i) aged from 20 to 64, (ii) economically active in the Korean labor market, and (iii) waged employees.

### Characteristics

We defined full-time, permanent employees as standard employees, and part-time, temporary, and daily employees as nonstandard employees [21–22]. "Full-time and permanent" were defined as working full-time with permanent contracts with a duration of over one year. "Part-time and permanent" were defined as working part-time with permanent contracts with a duration of over one year. "Temporary" was restricted to employees with contracts with a duration between one month and one year. "Daily" included employees with contracts with a duration of one month or less.

Sociodemographic characteristics included age, occupational class, monthly household income, educational level, and marital status. Occupational class was classified as either “manual” or “non-manual” work based on the Korean Standard Classification of Occupations [23]. Household income, which was defined as average monthly household income, was categorized into quartiles of “high,” “upper middle,” “lower middle,” and “low”. Educational level was classified into “university, college or higher,” “middle or high school,” and “elementary school or lower.” Marital status was divided into “married” and “others,” with the latter including single, bereaved, divorced, and separated. These variables were designated as covariates in the logistic regression analysis.

### Measures

The outcome measures used in this study included health risk behaviors, measured cardiovascular health status, and the use of preventive screening services. All data were obtained by self-administered questionnaires from KNHANES IV, with the exception of the measured variables. Tobacco consumption was classified into two groups: i) current smoker and those who have a lifetime consumption of over 100 cigarettes and ii) ex-smoker who did not fulfill the first criterion or never smoker [24]. The survey defined binge drinking as seven or more standard drinks on a single occasion for men and five or more for women. According to the physician’s screening guide of the U.S. National Institute on Alcohol Abuse and Alcoholism (NIAAA), a response of “1 or more binge drinking events in a week” was defined as high-risk alcohol use [25]. Physical inactivity was assessed with a questionnaire based on a shortened version of the International Physical Activity Questionnaire (IPAQ) [26]. People who did not meet physical activity level categories 2 (moderate) or 3 (high) were considered physically inactive. Short sleep duration was defined as daily sleep time of less than six hours [27].

Hypertension was defined as systolic blood pressure (SBP) ≥140 mmHg, diastolic blood pressure(DBP) ≥ 90 mmHg, or taking medication for hypertension, which is in accordance with the Seventh Report of the Joint National Committee on the Prevention, Detection, Evaluation, and Treatment of High Blood Pressure [28]. All of the biochemical metabolic markers were estimated from morning fasting blood samples. Diabetes mellitus was defined as serum fasting blood glucose ≥ 126 mg/dl or having a prior medical diagnosis of diabetes mellitus. The criteria for low-density lipoprotein (LDL), high-density lipoprotein (HDL), triglycerides, total cholesterol, and obesity based on BMI were derived from the National Cholesterol Education Program Adult Treatment Panel III. High LDL, high triglycerides, and high total cholesterol were defined as 130 mg/dl or more, 150 mg/dl or more, and 200 mg/dl or more in a fasting state, respectively. HDL levels of less than 40 mg/dl in men or 50 mg/dl in women were regarded as low. A BMI of 25 kg/m^2^ or higher was regarded as obese.

Participants were asked to answer several questions about the last regular check-up of blood pressure and serum glucose level; these questions were based on the recommended screening intervals from established guidelines on health screening services. The suggested screening intervals were two years [28] and three years [29] for blood pressure and serum glucose, respectively. Analyses were completed with dichotomous variables that assessed whether the respondents received the check-ups within the suggested intervals.

### Statistical analysis

All statistical analyses in this study were performed with the STATA 12.0 software (Stata Corp, College Station, TX, USA). We calculated the proportions of health outcomes and reported descriptive statistics for each response by gender and employment status. We used multivariate logistic regression adjusted for age, household income, education level, and region. We accounted for survey weights by calculating the adjusted odds ratios (aORs) and 95% confidential intervals (CIs).

## Results

### Sociodemographic characteristics of the sample population

Table 1 shows the demographic and socioeconomic profiles of the study population by employment type and gender. The proportion of nonstandard employees was higher in women than in men. Among married employees, a higher proportion of men (81.07%) were standard employees compared to women (61.24%). Both men and women nonstandard employees tended to earn less and be less educated compared to standard employees. Additionally, nonstandard employees were predominantly engaged in manual occupations in both genders (55.88% in men, 69.50% in women).

**Table 1:** Socio-demographic characteristics of the sample population.

Characteristics	Male		p-value^*^	Female		p-value^*^
	Standard	Non-standard		Standard	Non-standard	
	N (%)	N (%)		N (%)	N (%)	
	2,167(75.22)	714(24.78)		1,326(53.97)	1,131(46.03)	
**Age**			<0.001			<0.001
20–34	658(30.36)	238(33.33)		534(40.27)	303(26.79)	
35–44	827(38.16)	169(23.67)		417(31.45)	325(28.74)	
45–54	470(21.69)	145(20.31)		275(20.74)	321(28.38)	
55–64	212(9.78)	162(22.69)		100(7.54)	182(16.09)	
**Occupational class**			<0.001			<0.001
Nonmanual work^a^	1,473(68.10)	315(44.12)		832(62.75)	345(30.50)	
Manual work^b^	690(31.90)	399(55.88)		494(37.25)	786(69.50)	
**Monthly household income**			<0.001			<0.001
Q1 (High)	606(28.41)	89(12.81)		365(27.82)	175(15.65)	
Q2 (Upper middle)	655(30.71)	144(20.72)		366(27.90)	223(19.95)	
Q3 (Lower middle)	568(26.63)	179(25.76)		273(20.81)	279(24.96)	
Q4 (Low)	304(14.25)	283(40.72)		308(23.48)	441(39.45)	
**Education level**			<0.001			<0.001
University, college or higher	1,170(53.99)	135(18.93)		605(45.63)	261(23.08)	
Middle or high school	906(41.18)	454(63.67)		608(45.85)	615(54.38)	
Elementary school or lower	91(4.20)	124(17.39)		113(8.52)	255(22.55)	
**Marital status**			<0.001			0.061
Married	1,756(81.07)	434(60.87)		812(61.24)	734(64.90)	
Others^c^	411(18.93)	280(39.13)		514(38.76)	397(35.10)	

^*^ P-value obtained from analysis of Pearson's chi-square test between the two groups of standard and nonstandard workers

^a^ Nonmanual class included legislators, senior managers, administrators, professionals, technicians, paraprofessionals, and office workers

^b^ Manual class included service, sales, skilled agricultural, foresty, fishery workers, craft, related trades workers, plant, machine operators, assemblers, and unskilled labor

^c^ Non-married group included single, bereavement, divorcement, and living separately

### Differences in cardiovascular health risk behaviors between standard vs. nonstandard employees

Table 2 shows the prevalence of health risk behaviors. The crude proportions were calculated using the sampling weights, and aORs were calculated using logistic regression after adjusting for demographic and socioeconomic factors. The rates of current cigarette smoking and high-risk alcohol drinking were generally higher in nonstandard employees in both genders, although male standard employees were more likely to engage in high-risk alcohol consumption than were male nonstandard employees.

**Table 2:** Differences in the cardiovascular health risk behaviors between standard and nonstandard employees.

Measure	Male		95% CI for aOR	Female		95% CI for aOR
	Standard	Non-standard		Standard	Non-standard	
	2,167(75.22)	714(24.78)		1,326(53.97)	1,131(46.03)	
**Cigarette smoking**^**a**^						
Crude proportion (%)	49.1	54.97		6.71	9.13	
aOR^*^	1	1.26	1.01–1.59	1	1.61	1.10–2.35
aOR^**^	1	0.92	0.73–1.15	1	1.12	0.74–1.70
**High-risk alcohol use**^**b**^						
Crude proportion (%)	33.93	30.63		9.36	11.7	
aOR^*^	1	0.86	0.67–1.09	1	1.59	1.10–2.29
aOR^**^	1	0.8	0.62–1.03	1	1.12	0.77–1.64
**Physical inactivity**^**c**^						
Crude proportion (%)	39.33	36.35		43.77	41.85	
aOR^*^	1	0.88	0.70–1.11	1	0.92	0.74–1.13
aOR^**^	1	0.95	0.74–1.21	1	0.94	0.76–1.16

^*^ Adjusted using logistic regression to control for age

^**^ Adjusted using logistic regression to control for age, household income, education level, occupational class (manual or nonmanual), region

^a^ Current smoker with the amount of life-time smoking ≥ 100 cigarettes

^b^ Drinking more than 7drinks in men and 5 drinks in women on ≥ 1 occasion in an average week

^c^ Not engaged in either 20 min (or more) of vigorous physical activities, three or more times per week, or 30 min (or more) of light/moderate physical activities, five or more times per week

### Differences in measured cardiovascular morbidities between standard vs. nonstandard employees

Table 3 shows the distribution of measured variables, including hypertension, diabetes mellitus, lipid abnormalities, and obesity. For hypertension, crude proportions were higher in female nonstandard employees, and the aOR of hypertension (aOR 1.42, 95% CI 1.02 to 1.98) was elevated compared to that of standard employees. Furthermore, female nonstandard employees showed higher rates of high LDL, total cholesterol, triglycerides, and low HDL. On the other hand, male nonstandard employees showed lower prevalence of elevated LDL, total cholesterol, triglycerides, as well as lower prevalence of low HDL. Female nonstandard employees showed higher prevalence of obesity than male nonstandard employees.

**Table 3:** Differences in the measured cardiovascular morbidities between the standard and non-standard employees.

Measure	Male		95% CI for aOR	Female		95% CI for aOR
	Standard	Non-standard		Standard	Non-standard	
	2,167(75.22)	714(24.78)		1,326(53.97)	1,131(46.03)	
**Hypertension**^**a**^						
Crude proportion (%)	23.08	21.76		8.52	17.09	
aOR^*^	1	0.82	0.63–1.07	1	1.46	1.06–2.01
aOR^**^	1	0.83	0.62–1.09	1	1.42	1.02–1.98
**Diabetes**^**b**^						
Crude proportion (%)	6.05	7.69		2.38	5.3	
aOR^*^	1	1.03	0.67–1.58	1	1.44	0.85–2.44
aOR^**^	1	1.17	0.73–1.90	1	1.14	0.66–1.98
**High LDL** ^**c**^						
Crude proportion (%)	24.75	21.46		18.12	24.05	
aOR^*^	1	0.82	0.62–1.09	1	1.08	0.83–1.41
aOR^**^	1	0.86	0.63–1.17	1	1.1	0.84–1.44
**Low HDL** ^**d**^						
Crude proportion (%)	30.81	33.77		35.93	44.26	
aOR^*^	1	1.15	0.88–1.49	1	1.28	1.01–1.63
aOR^**^	1	1.29	0.97–1.71	1	1.25	0.99–1.59
**High triglycerides**^**e**^						
Crude proportion (%)	36.67	34.69		11.38	18.44	
aOR^*^	1	0.91	0.71–1.17	1	1.43	1.05–1.95
aOR^**^	1	0.96	0.72–1.28	1	1.36	0.99–1.87
**High total choldesterol**^**f**^						
Crude proportion (%)	34.54	28.7		23.58	29.22	
aOR^*^	1	0.75	0.59–0.95	1	1.02	0.80–1.30
aOR^**^	1	0.82	0.64–1.04	1	1	0.78–1.29
**Obesity**^**g**^						
Crude proportion (%)	38.66	32.85		17.88	24.52	
aOR^*^	1	0.77	0.62–0.97	1	1.21	0.94–1.56
aOR^**^	1	0.89	0.70–1.13	1	1.11	0.86–1.43

^*^ Adjusted using logistic regression to control for age

^**^ Adjusted using logistic regression to control for age, household income, education level, occupational class (manual or nonmanual), region

^a^ SBP ≥ 140 mm Hg or a DBP ≥ 90 mm Hg or being treated with anti-hypertensive medication

^b^ Fasting plasma glucose ≥ 126 mg/dL or with prior medical diagnosis of diabetes mellitus

^c^ Fasting LDL ≥ 130mg/dL

^d^ Fasting HDL <40 mg/dL in male, and < 50 mg/dL in female

^e^ Fasting triglycerides ≥ 150 mg/dL

^f^ Fasting total cholesterol ≥ 200mg/dL

^g^ BMI ≥ 25 Kg/m2 calculated from anthropometric measurements

### Differences in the use of preventive health screening services between standard and nonstandard employees

Table 4 shows the differences in the use of preventive health screenings comparing standard to nonstandard employees. The rates of hypertension and diabetes mellitus screening were lower among nonstandard workers for both men (aOR 0.72, 95% CI 0.54 to 0.94 in hypertension screening, aOR 0.58, 95% CI 0.43 to 0.79 in diabetes mellitus screening) and women (aOR 0.56, 95% CI 0.43 to 0.73 in hypertension screening, aOR 0.55, 95% CI 0.43 to 0.71 in diabetes mellitus screening).

**Table 4:** Differences in the use of cardiovascular health screening services between standard and non-standard employees.

Measure	Male		95% CI for aOR	Female		95% CI for aOR
	Standard	Non-standard		Standard	Non-standard	
	2,167(75.22)	714(24.78)		1,326(53.97)	1,131(46.03)	
**HT screening**^**a**^						
Crude proportion (%)	84	72		80.53	68.83	
aOR^*^	1	0.51	0.39–0.65	1	0.48	0.37–0.62
aOR^**^	1	0.72	0.54–0.94	1	0.56	0.43–0.73
**DM screening**^**b**^						
Crude proportion (%)	83.47	66.6		77.64	66.55	
aOR^*^	1	0.4	0.31–0.53	1	0.50	0.39–0.64
aOR^**^	1	0.58	0.43–0.79	1	0.55	0.43–0.71

^*^ Adjusted using logistic regression to control for age

^**^ Adjusted using logistic regression to control for age, household income, education level, occupational class (manual or nonmanual), region

^a^ Receipt of the screening of blood pressure within past 2 years

^b^ Receipt of the screening of plasma glucose level within past 3 years

## Discussion

### Principal findings

Both male and female nonstandard employees were less likely to receive preventive screening services for hypertension and diabetes compared to standard employees. Furthermore, we found that female nonstandard employees tended to have higher blood pressure than did female standard employees.

Job security only represents part of the psychosocial work environment. Therefore, factors such as occupational categories, increased workload and decreased job control (i.e., skill discretion and opportunities to participate in decision making) could partly mediate the effect of job insecurity on cardiovascular health status [30–31]. Although these factors are not represented in the national surveys, such additional factors may explain why women in nonstandard employment had higher prevalence of hypertension compared to that of standard female employees. Therefore, a multifactorial assessment evaluating the underlying effect of job insecurity on cardiovascular health disease is needed in the near future.

### Policy implication of the study

The prevention of cardiovascular disease is dependent on the successful control of risk factors, such as high blood pressure, serum glucose levels, and serum cholesterol levels [32]. Since early detection and treatment of hypertension are required to prevent cardiovascular diseases, international guidelines typically emphasize the importance of medical health screening, risk assessment, and early intervention [33]. Although cardiovascular disease detection and prevention have had a significant impact on public health [34], there has been little practical effort to remediate the access issue for nonstandard employees. This is important since prolonged activation of the stress system due to employment insecurity may induce cardiovascular, immune, metabolic, hemostatic and other adverse changes in health behavior [35]. Additionally, it has been widely recognized that health risk behaviors and measured morbidities are linked to long-term morbidity and mortality [36]. Therefore, it is imperative to focus on cardiovascular illnesses of nonstandard employees to prevent the potentially detrimental health impacts of nonstandard employment. If Korean health systems continue to not accommodate for nonstandard employees, the health status of disadvantaged employees will likely further decline.

### Weaknesses of the study

Our study has several limitations. First, due to the fact that our design is cross-sectional, the direction of causation could not be clearly shown. For example, the “healthy worker effect” operates so that individuals with health problems are more likely to be selected into nonstandard jobs, and thus there is the possibility of reverse causation [37–38]. Second, our analyses were partly based on self-reported data and therefore subject to information bias [39]. Third, there is an ongoing debate about the definition of nonstandard work and similar labor market arrangements even in European countries where these arrangements have existed for longer durations [40]. Therefore, the standardized criteria for nonstandard employment are needed for the purpose of comparison on an international scale. For example, while U.S. studies include nonstandard individuals who are self-employed (e.g. independent contractors, who represent about 7% of the work force—Kalleberg, Annu Rev Sociol (2000)) as nonstandard employees, these individuals were not included in our definition. Finally, unmeasured personal characteristics, work-related factors, and other confounders may have contributed to the association between work strain and certain health risk factors.

Despite the aforementioned limitations, our representative national data allowed us to estimate job-security-related disparities in preventive screening services between standard vs. nonstandard employees. More effort is needed to enable nonstandard employees to access and receive regular health screening services based on the social insurance system. To reduce the disparities between standard and nonstandard employees, further attention and effective collaboration involving policymakers, health plan administrators, third-party payers, and health care providers are needed.
